# A Vehicle Recognition Algorithm Based on Deep Transfer Learning with a Multiple Feature Subspace Distribution

**DOI:** 10.3390/s18124109

**Published:** 2018-11-23

**Authors:** Hai Wang, Yijie Yu, Yingfeng Cai, Long Chen, Xiaobo Chen

**Affiliations:** 1 School of Automotive and Traffic Engineering, Jiangsu University, Zhenjiang 212013, China; wanghai1019@163.com (H.W.); 2221704084@stmail.ujs.edu.cn (Y.Y.); 2 Automotive Engineering Research Institute, Jiangsu University, Zhenjiang 212013, China; chenlong@ujs.edu.cn (L.C.); xbchen82@gmail.com (X.C.)

**Keywords:** vehicle recognition, deep transfer learning, multiple subspace feature distribution, intelligent vehicles

## Abstract

Vehicle detection is a key component of environmental sensing systems for Intelligent Vehicles (IVs). The traditional shallow model and offline learning-based vehicle detection method are not able to satisfy the real-world challenges of environmental complexity and scene dynamics. Focusing on these problems, this work proposes a vehicle detection algorithm based on a multiple feature subspace distribution deep model with online transfer learning. Based on the multiple feature subspace distribution hypothesis, a deep model is established in which multiple Restricted Boltzmann Machines (RBMs) construct the lower layers and a Deep Belief Network (DBN) composes the superstructure. For this deep model, an unsupervised feature extraction method is applied, which is based on sparse constraints. Then, a transfer learning method with online sample generation is proposed based on the deep model. Finally, the entire classifier is retrained online with supervised learning. The experiment is actuated using the KITTI road image datasets. The performance of the proposed method is compared with many state-of-the-art methods and it is demonstrated that the proposed deep transfer learning-based algorithm outperformed existing state-of-the-art methods.

## 1. Introduction

With rapid economic and technological progress, the development of modern transportation tools, such as transportation vehicles, satisfactorily facilitate the requirements of life and work. Meanwhile, rising vehicle use causes social problems such as accidents, traffic congestion, and consequent traffic pollution. Therefore, the way these problems are solved, while also retaining the benefits of these tools, has become a worldwide problem.

Recently, as an important part of Intelligent Transportation Systems (ITS), Intelligent Vehicles (IVs) have drawn increasing attention from researchers and industry as potential solutions to mitigate these problems [1]. IVs are expected to possess both high environmental sensing ability and a high intelligence, which is in reality an intelligent agent containing an environmental perception layer, a decision planning layer, and an operation control layer.

The IV environmental perception layer obtains road environment information through different sensors to achieve detection and tracking of surrounding obstacles such as road structures, vehicles, pedestrians, road lanes, traffic signs, and traffic signals. It provides critical information for the decision planning and operation control layers. Therefore, its performance directly affects the overall function of the intelligent vehicle.

Other surrounding vehicles are the most common obstacles for IVs, which are critical obstacles when considering road security. However, another major performance impediment for IVs is a low detection rate for other vehicles. There are two reasons that lead to this issue. First, vehicle detection must occur in a very complex road environment scene. The camera in the ego-vehicle (i.e., “our” car) and the target vehicles are both in motion. As a result, the visual information about the target vehicles is easily contaminated or occluded by other objects. Secondly, the visual information itself is also very complex. There are thousands of types of vehicles in the world, and each type of vehicle can have many different possible appearances (e.g., colors and trim styles.)

**Figure 1:**
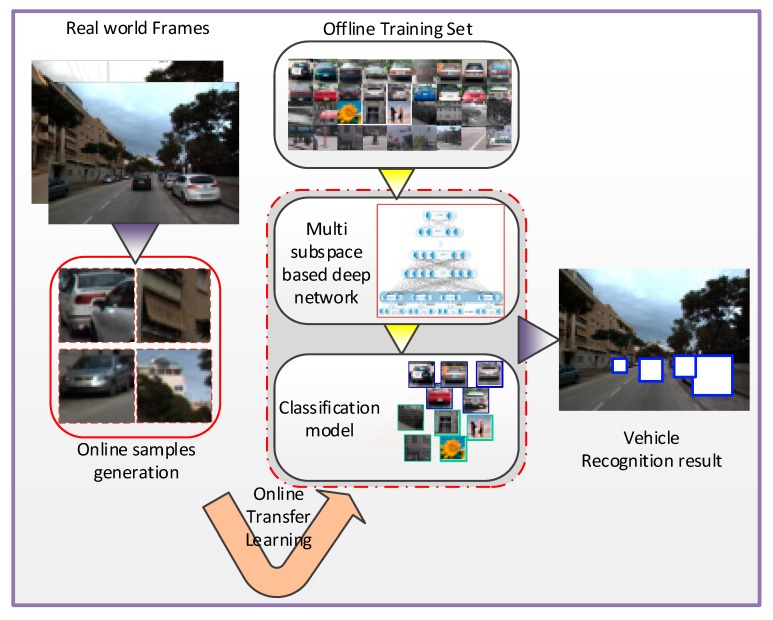
Overall framework of the proposed algorithm.

In this work, a novel vehicle classifier based on deep learning and transfer learning is proposed which focuses on solving the two deficiencies mentioned above. First, the deep learning framework is induced in vehicle detection tasks in which a deep network based on the multiple independent feature subspace distribution assumption being established. Second, transfer learning theory is applied for online classifier retraining, and the retraining algorithm is proposed based on the deep model that was built. The overall framework is shown in Figure 1.

The article will be divided as follows: in Section 2, related work will be described. In Section 3, the proposed work will be introduced in detail. In Section 4, the experiments will be given. Finally, conclusions will be presented in Section 5.

## 2. Related Work

Early work on vehicle detection for detecting vehicles mainly focused on simple features with clear physical meanings, such as symmetry, edges, underbody shadows, textures, and corners [2]. This kind of method can be characterized by its low environmental adaptability, and the methods often fail in complex backgrounds or under changes in illumination. Motion-based methods are another popular framework for vehicle detection, however poor performance is demonstrated when the ego-vehicle and target vehicle are experiencing relatively small movement [3]. Yet another approach, template matching, is an additional way for vehicle detection of fully visible vehicles, however, the method cannot handle occluded conditions [4]. 

With recent developments in the areas of machine learning and statistical theory, researchers have found that the image detection problem should be modelled as a two-class classification issue [5]. Under this approach, vehicle detection capabilities have made tremendous progress, and the two-class approach has become the most popular choices for vehicle detection applications. In a two-class classification framework, there are two critical steps: (1) extracting the appropriate representation features for vehicle images; and, (2) constructing, training, and optimizing the vehicle classifier. Of the two steps, vehicle feature extraction is arguably more important. Effective feature extraction can reduce the dependency on further classifier learning algorithms and improves the performance of the whole system [6].

For vehicle feature representation, the most common features are Histogram Orientation Gradients (HOG) features designed by Dalal [7], Haar features designed by Papageorgiou [8], and LBP features [9]. Some deformation features based on these three kinds of features are also used in vehicle detection applications. For example, Cheon proposed a symmetry HOG feature [10], Pham proposed polygon Haar features [11], and Mohamed proposed a fast Haar feature [12]. Furthermore, other features such as SIFT and SURF [13], and subspace extraction methods such as PCA and ICA [14], are also used in this application. These features can be further integrated with classifiers such as SVM, boosting, and neural networks to archive full classifier training [15,16]. Recently, a framework named deep learning has been increasingly used for object detection or classification by researchers. Compared with the shallow model, the deep mode has more power in feature extraction and complex classification function building ability. Deep Convolution Neural Network (DCNN) and Deep Belief Network (DBN) are the two most popular deep models which were proposed by Lecun and Hinton [17,18]. Inspired by the good performance of these two deep models, many new deep architectures have been proposed such as VGG [19], GoogleNet [20], ResNet [21], Faster R-CNN [22], SSD [23] and YOLO [24]. 

For better understanding, the pros and cons of related work and the proposed method in this work are compared and summarized in Table 1. In general, although the above-mentioned research efforts have achieved increased vehicle detection accuracy, most of the existing methods are still not able meet the full requirements for accurate vehicle detection in real, complex, and dynamic traffic environments, such as mixed traffic environments with vehicles, bicycles, and pedestrians, which are common in most parts of China. Through analysis, it can be concluded that existing work suffers from two fundamental deficiencies:

(1) The abilities of hand-crafted features and complex function descriptions of the shallow models are insufficient for vehicle classification in complex traffic environments. In addition, for the deep learning framework, it is still necessary to figure out how to find a better deep structure model.
(2) Vehicle classifiers that are only trained with offline samples are not suitable for the enormous diversity and dynamic nature of actual traffic scenes. Vehicle detection is often applied on moving platforms, which must rely on different kinds of scenes, such as downtown and urban landscapes. The classifiers must also work under different illumination levels, such as daytime, night, or evening. Therefore, if the distribution of actual samples in a real traffic scene contains a big difference of training samples, classification performance drops dramatically.

## 3. Proposed Model

**Figure 2:**
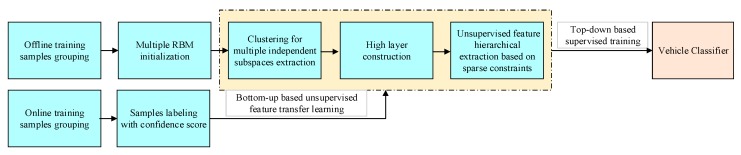
Algorithm flow chart.

As shown the flow chart in Figure 2 below, the proposed methods are divided into two main parts. The first part is offline training step based on an improved deep belief network (DBN) based in which multiple Restricted Boltzmann Machines (RBMs) is used to extract multiple independent feature subspace distribution in the lower layer. This part will be detailed introduced in Section 3.1. The second part is online transfer learning step in which new training samples will be generated and labeled with a confidence score online and the whole networks will be retrained. This second part will be detailed introduced in Section 3.2.

### 3.1. Deep Model Construction and Feature Extraction Based on Feature Distribution in Multiple Independent Subspaces

In traditional research, features are considered to be attached on a complex space. However, images are very high-dimension data which are difficult to express fully in one isolated space. A more reasonable assumption is that image features are distributed among several independent nonlinear subspaces [25]. A corresponding deep model is established based on this assumption and a sparse constraint-based unsupervised feature extraction algorithm is also developed.

#### 3.1.1. Lower Layer Design of Deep Network and Multiple Independent Subspaces Extraction

**Figure 3:**
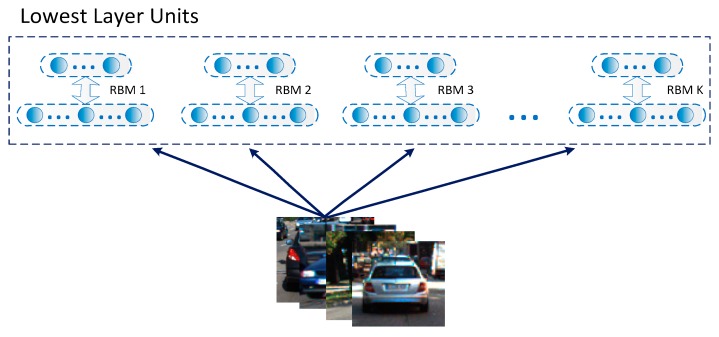
Design of lower-layer units in the designed deep model.

Deep networks with multiple layers generally exhibit excellent feature learning capabilities. Among many constituent units of deep networks, Restricted Boltzmann Machines (RBMs) is a typical example. The RMB is a simplified and fully connected Boltzmann machine in which units in each layer are independent. The RBM is actually an energy model, meaning it is modeled as a parametric model in order to characterize a probability distribution, and it is able to train linear subspace distributions based on energy. Compared with other nonlinear subspace learning methods, there is no need for RBMs to use preset parameters such as dimension or complexity. So, the RBM is very suitable as a generic subspace learning machine. Given the analysis above, the RBM is used in this work to form lower-layer units of the designed deep model to achieve multiple subspace extractions, as shown in Figure 3.

**Figure 4:**
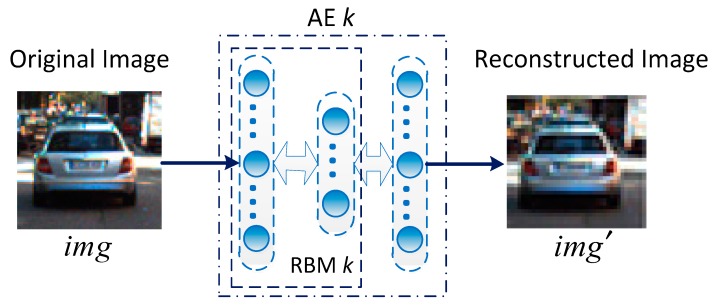
Feature subspace clustering and samples reconstruction based on auto-encoder.

For multiple subspace extraction algorithms, one independent subspace is learned in each branch of the proposed *K* RBMs. In each RBM, *I* visible units are equal to the dimensions of the image data and the hidden layers are grouped with *J* hidden units, corresponding to the dimensions of the input image data. In unsupervised feature training, a group of new units is added to each RBM and its dimension is equal to that of the visible layer of each RBM. With this structure, *K* RBMs are formed as *K* Auto-Encoders. Then, every sample without label information is input to each Auto-Encoder to calculate the reconstruction error. If the *K*-th RBM subspace has the smallest reconstruction error min(|img−img′|), it is clustered with the *K*-th RBM unit (Figure 4). Finally, a Contrastive Divergence algorithm is used to iteratively update the RBM weights by loading all the samples [26]. 

Based on the steps above, each nonlinear feature subspace can be extracted by clustering all image samples and updating the RBM weights.

#### 3.1.2. High Layer Construction

**Figure 5:**
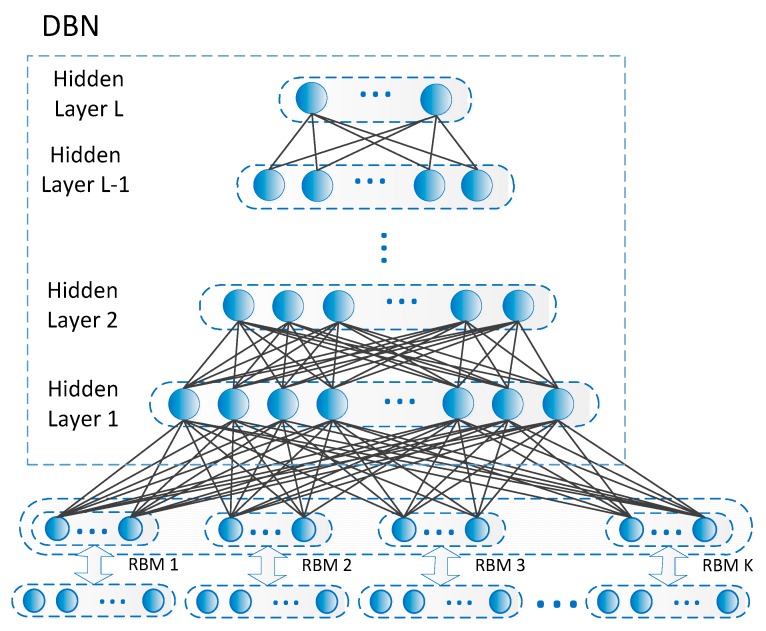
Upper structure design based on DBN.

Through reconstruction clustering and weight updates, each feature in the multiple nonlinear subspaces is extracted for each RBM. Then, those low-level features must be further extracted to achieve high-level semantic feature generation. From there, a multi-layer DBN is selected to form the upper network structure of the proposed deep model (Figure 5). DBN is a probabilistic model composed of multiple layers of stochastic, hidden variables. A typical DBN is with one input layer $V_{1}$ and L hidden layers $H_{1}$, $H_{2}$ …$H_{L}$ while x is the input data which can be for example a vector, and y is the learning target e.g., class labels. Here, in this application, the lowest layer of the DBM is fully connected with multiple RBMs. With the hidden layers in DBNs, the lower-layer features in the RBM can be further extracted in an unsupervised manner.

**Figure 6:**
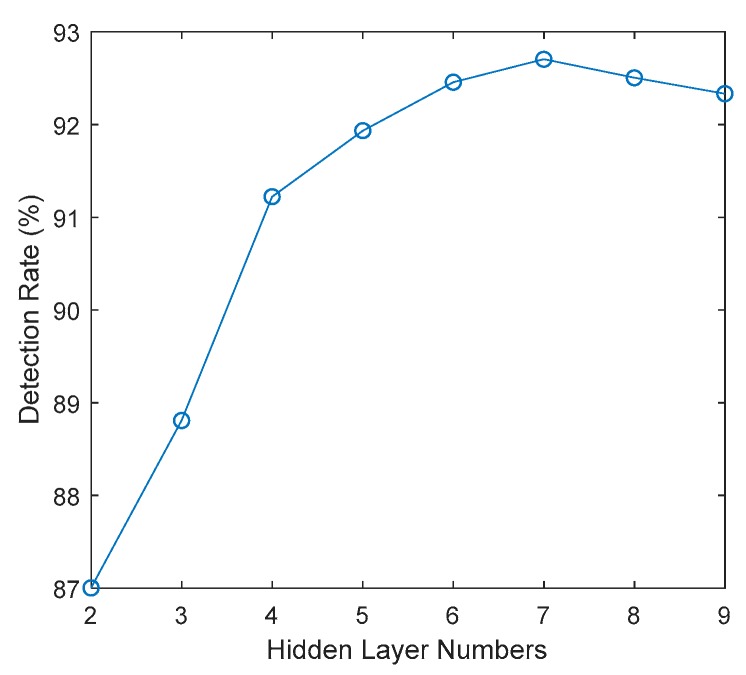
Hidden layer numbers for DBN.

For the setting of hidden layers number. A group of numbers are tested in which 7 hidden numbers are with the best detection rate. As shown in Figure 6.

#### 3.1.3. Unsupervised Feature Hierarchical Extraction Based on Sparse Constraints

Cognitive scientists have found that the human brain processes visual information through a bottom-up, layer-by-layer signal extraction process, and converts visual signals to semantic information. Inspired by this, a sparse constraint-based unsupervised hierarchical feature extraction method is proposed.

Greedy layer-by-layer reconstruction algorithms are normally used in traditional training methods to update weights between adjacent layers. Let us take feature layer $V_{1}$ and hidden layer $H_{1}$ as an example. The training target function, which is also the joint probability distribution of input state $v^{1}$ and hidden state $h^{1}$, is written as:

(1) $$E_{DBN}=p(v)=\frac{1}{Z}\sum_{h}\exp(-E(v,h))\;$$

in which, $Z=\sum_{v}\sum_{h}\exp(-E(v,h))$

In this work, a sparse constraint in L1 norm like (2) is added to the original target function. It constructs a sparser and structured weights pattern to achieve a more expressive feature extraction:

(2) $$\sum_{l=1}^{N}||p(h^{\left(l\right)}|v^{\left(l\right)})|{|}_{1}\;$$

In the above, l=1,2…N are all the training samples for *N* total training samples. Given the sparse constraint function, the new target function $E_{S-DBN}$ can be written as:

(3) $$E_{S-DBN}=(1-\lambda )p(v)+\lambda \sum_{l=1}^{N}||p(h^{\left(l\right)}|v^{\left(l\right)})|{|}_{1}\;$$

in which λ=0.3 is given as a regularization weighting factor.

### 3.2. Classifier Transfer Learning Combined with Top-Down and Bottom-Up Framework

Traditional two-class classification-based methods hold the assumption that training samples and test samples are both independent but have the same distribution. In our vehicle detection tasks, the on-board camera is always moving, and the captured traffic images contain dynamic, random characteristics due to weather, illumination level, and traffic variety. Therefore, in this application, the distributions of the training and testing samples are different and will therefore not satisfy the assumption of similarly-shaped distributions. As a result, the classifier classification ability would drop in real-world applications. To avoid this potential failure, a sample labelling method in dynamic scenes and an online transfer training method based on the given deep model are also proposed in this work.

#### 3.2.1. Sample Labeling Method in New Scenes

Sample generation and labelling is the first step for transfer learning. Existing methods often employ man-made sample generation and labelling, which are not suitable for the automation requirements of this task. As an alternative, computer-based sample labelling maintains label tag uncertainties. To solve this, a novel sample selection and labelling method with tag confidence is proposed based on Bagging (Bootstrap aggregating) ensemble learning.

**Figure 7:**
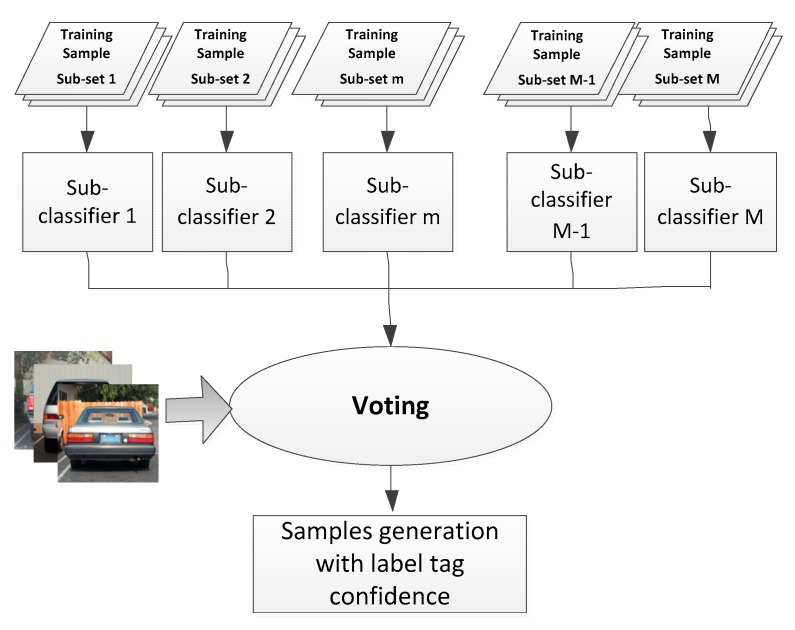
Sample label tag generation with confidence score.

Here, several separate sub-training datasets are first prepared, and each sub-classifier is trained on a single dataset only (Figure 7). Using a voting mechanism, the final output of each classifier is decided by each sub-classifier. A few relatively independent source training data sets, ${ϒ}_{m}$ (m=1,…,M, where M is the number of source training data sets), are prepared. All the training data sets are isolated which are captured under different weather conditions, different scenarios, and even different camera equipment. Each vehicle classifier, $\Phi_{m}$, is trained using a single independent source training data set, ${ϒ}_{m}$. The confidence score, *S*, of this sample is calculated with Equation (4):

(4) $$s=\hat{m}/M\;$$

In this application, our group selects the M=9 independent dataset for sub-classifier training.

#### 3.2.2. Bottom-Up Based Unsupervised Feature Transfer Learning

In a deep learning framework, features are extracted from the general, in the lower layers, to the specific in the higher layers. Specifically, the features extracted in higher layers are very close to the classification tasks and the transfer ability is relatively small compared to lower layer features. For this reason, a transfer learning algorithm is proposed, which first transfers low layer features and then transfers the high layer features.

In low-level feature transfer learning, RBM is also used as a subspace unit and new samples are clustered in each RBM to make the feature transfer. The clustering and layer-by-layer training method is the same as the method described in Section 3.1.

#### 3.2.3. Top-Down Based Supervised Deep Network Training

In top-down based supervised deep network training, the training is performed between the highest layer and the label layer, as described below. It is defined that the feature in the highest layer of the nth newly generated sample is $f_{n}$, and its label is $y_{n}$. Then, the parameter set of this sample is written as $\left\{f_{n},y_{n},s_{n}\right\}$, in which $s_{n}$ is the sample label tag confidence. A new training target function based on these settings is defined as:

(5) $$Loss=\sum_{n}s_{n}Loss^{E}(y_{n},{\tilde{y}}_{n})\;$$

In this target function, $Loss^{E}(y_{n},{\tilde{y}}_{n})=-y_{n}\log{\tilde{y}}_{n}-(1-y_{n})\log(1-{\tilde{y}}_{n})$ is a cross-entropy loss function to estimate the difference between the estimated label and the true label, while $s_{n}$ gives the corresponding weight between each difference. In training, a Back Propagation (BP) algorithm is used to both optimize and minimize the target function to update the network weights.

## 4. Experiment and Analysis

### 4.1. KITTI Vehicle Dataset

In this paper, the experimental images are derived from the KITTI standard road image data base. This database provides images taken under various road conditions and provides an accurate annotation [27] of road objects (including vehicles). The KITTI road image data base is randomly divided into two parts, including a training set and a test set. The KITTI training set contains 7481 pictures, and includes 35,000 vehicles, while the KITTI test set contains 7518 pictures, and includes approximately 27,000 vehicles.

In the experiment, offline training positive samples come from the 9 independent samples set described in Section 3.2, as well as other datasets, such as the Caltech99 and Malaga datasets. All vehicle samples are integrated into a large positive sample library, which contains 18,000 vehicles. The images for training sample generation in the target scene come from the KITTI training set, and the new samples with label confidence are generated with the method described in Section 3.2. All the negative training samples are generated from 20,000 images of the KITTI training set, which do not contain vehicles. The test sets are grouped with 2000 road images randomly selected from the KITTI test set containing 7218 vehicles. The experiment dataset details is shown in Table 2.

### 4.2. Experiment

In this section, two groups of experiments were designed. Experiment one was a classifier performance comparison experiment without the introduction of transfer learning. In this experiment, all the classifiers were trained with offline samples and the performance of the multiple subspace-based deep classifier proposed in this work is compared to multiple classification algorithms. The second experiment compares the proposed deep model and transfer-learning based classifier to a few of the existing state-of-the-art transfer learning classifiers. The test sets of the two groups were all KITTI vehicle data sets. In the test data set, each sub image of KITTI dataset was input and judged by the classifier. The way of selecting sub images was by using traverse searching of the entire image from 24 × 24 pixels with a zooming scale of 1.1. For the sub image that is recognized as a vehicle by the classifier, if its test box has an 80% overlap with the box of ground truth, it was considered as a successful detection. By this metric, an ROC curve was used to evaluate the performance of each vehicle detection method. The experimental platform is as follows: processor: Intel Xeon E5-2687W V4 @3.00 GHZ; OS: Ubuntu16.04; memory: 128 G; graphics card: NVIDIA Quadro M4000. The Keras platform is used for training and running deep learning methods.

#### 4.2.1. Experiment 1

In this experiment, the performance of the multiple feature subspace deep model vehicle detection method was compared to several existing methods, including shallow model-based and deep learning-based methods. Here, the shallow model-based method included the Cascaded Adaboost method [28], and the deep learning-based method included ConvNet proposed in [29], Deep Convolutional Neural Networks (DCNN) [30], VGG [21], YOLOv3 [31] and Fast R-CNN [24]. The subspace numbers, which are also the RBM numbers, were separately set at 5, 10, 20, and 30, for comparison. Here, all these algorithms are set with their original settings and were trained with the same offline training samples and tested in the KITTI dataset.

**Figure 8:**
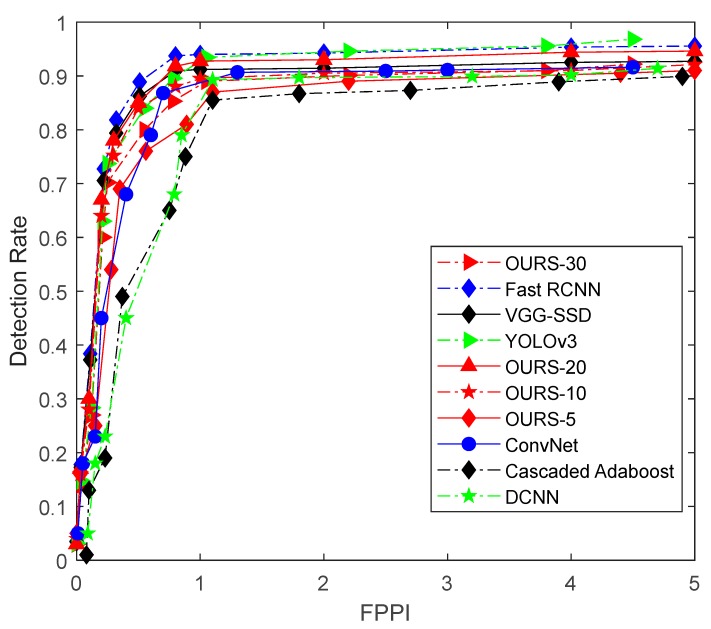
ROC curves for the performance of several offline learning algorithms using the KITTI test data set.

The experimental results are shown in the ROC curve in Figure 7 where the horizontal axis provides the number of False Positive Per Image (FPPI) and the vertical axis shows the detection rate. Here, OURS-5, OURS-10, OURS-20, and OURS-30 denote the classifier, in which the number of subspaces is 5, 10, 20, and 30, respectively. From the ROC curve in Figure 8, it is shown that the proposed deep vehicle classifier achieved the best vehicle detection rate for a subspace number set to 20. Meanwhile, when FPPI was equal to 1, the detection rate of our method OURS-20, VGG-SSD [21], Fast RCNN [24], Cascaded Adaboost [28], ConvNet [29], DCNN [30] and YOLOv3 [31] were 92.75%, 91.10%, 94.20%, 79.50%, 87.58%, 85.75% and 93.35%, respectively. Here, Fast R-CNN had the best performance in the non-transfer experiment and YOLOv3 and OURS-20 had the second-best performance. The reason for these results might be due to the advantages of the DCNN structure of image representation compared to the DBN structure. The training time for the proposed method is around 2.5 h in our experimental platform. Besides, the average processing time for the seven methods (OURS-20, VGG-SSD, Fast R-CNN, Cascaded Adaboost, ConvNet, DCNN, YOLOv3) are 175.8 ms, 963.1 ms, 562.6 ms, 33.9 ms, 80.6 ms, 139.5 ms and 104.7 ms for an input image of 1920 × 1280 scale without code optimization and acceleration.

#### 4.2.2. Experiment 2

In this experiment, the proposed multiple feature subspace distribution deep model and transfer learning-based method were compared with several transfer learning-based methods, such as the Confidence-Encoded SVM based method [32] and the ITL-AdaBoost based method [33]. In this experiment, the RBM number for our model was set at 20.

**Figure 9:**
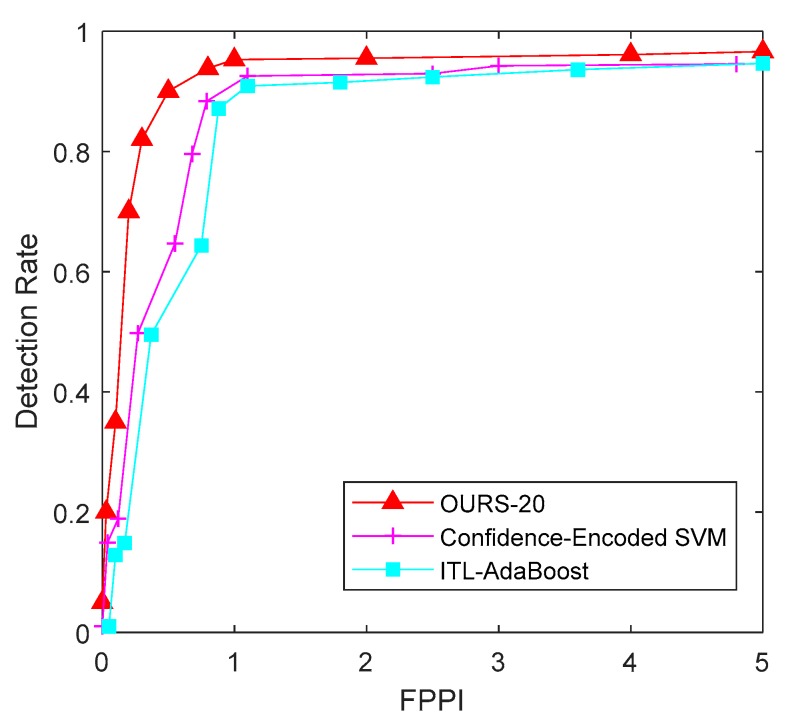
ROC curves of different algorithms under KITTI data set.

**Figure 10:**
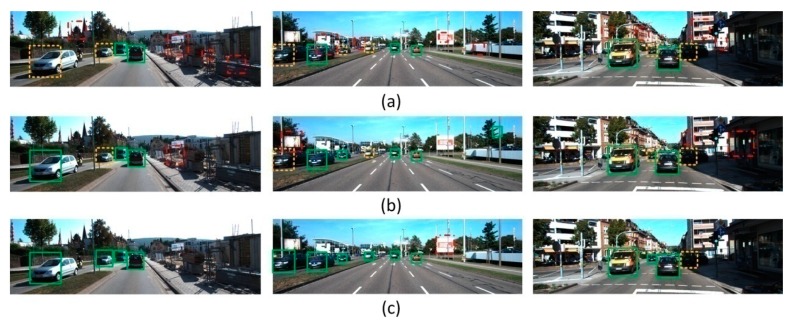
Example test results for different methods using the KITTI test data set. (**a**) Test results of ITL-Adaboost; (**b**) Test results of Confidence-Encoded SVM; (**c**) Test results of our method.

The ROC curve in Figure 9 shows that that for a FPPI equal to 1, the detection rate of our method, article [32], and article [33] were 95.36%, 92.82%, and 90.40%, respectively. It also shows that, because of the addition of transfer learning, the average overall detection rate rose dramatically, compared to the performance demonstrated in the first experiment. Figure 10 demonstrates the detection performance results of the three methods, in which sub-figures (a), (b), and (c) are the detection results of article [33], article [32] and our algorithm against the KITTI test images. Here, a green box means a correctly-detected vehicle, a yellow box means a miss detected vehicle (i.e., a vehicle is not detected), and a red box means a falsely detected vehicle (i.e., a detection is declared on something that is not a vehicle).

### 4.3. Experiment Analysis

Generally, compared with existed shallow model based transfer learning-based methods (Confidence-Encoded SVM and ITL-AdaBoost), it is found that most of the shallow model based transfer learning-based vehicle detection algorithms performed well on the easy objects, but there was a large difference in the detection precision for the moderate and hard objects with the scale transformation and the occlusion impact. Overall, the proposed methods is with significant improvement. However, in the non-transfer experiment, compare with existed deep model such as SSD and YOLOv3, the proposed method have less detection accuracy. In general, the proposed method still have some cons. 1. The processing time is relatively long since multiple RBN for subspace extraction and an extra online transfer process is added. 2. The number of subspaces is hard to determine with mathematical model, so at this stage we are still using multiple attempts to find the best number.

In future work, since the DCNN structure is more suitable for image representation, as shown in the experiment, we would like to work on establishing a multiple subspace DCNN structure-based transfer learning method to see if it is able to improve the performance. Besides, some code optimization and acceleration method such as parallel computing will be tried to use to boost the processing process.

## 5. Conclusions

This work proposes a vehicle detection algorithm based on a multiple subspace feature distribution deep model with online transfer learning. First, a deep model is established in which multiple RBMs are used to construct lower-layer multiple subspace features and a DBN is used to construct a superstructure. Then, for this deep model, an unsupervised feature extraction method is applied, based on sparse constraints. Second, a transfer learning framework with an online sample generation step is proposed, and the corresponding training method is given based on the deep model. Finally, an experiment using the KITTI dataset demonstrated that the proposed deep transfer learning method was better than many of the state-of-the-art transfer learning methods.

## Figures and Tables

**Table 1:** Pros and cons of related work and proposed method.

Methods	Pros	Cons
Simple features based methods such as symmetry, edges, underbody shadows, textures, and corners [2,3,4]	Easy to describe and perform in specific applications	Just can be used in specific very simple scene such as highway in good illumination, without the ability to other scene.
Manually feature and shallow learning model based methods [5,6,7,8,9,10,11,12,13,14,15,16]	Better detection and classification ability than simple features based methods, low training time and low resource requirement.	Still low detection performance in complex scene, without the ability to handle heavy occlusion
Deep model based methods [17,18,19,20,21,22,23,24]	Dramatically improved performance in vehicle detection and classification.	High training time and high resource requirement, classification performance drops dramatically when real traffic scene with big difference of training samples
The proposed method in this work	Better performance when real traffic scene with big difference of training samples	Lower real-time performance since multiple RBN for subspace extraction and an extra online transfer process is added.

**Table 2:** Experiment dataset details.

Samples Category	Source Dataset	Total Numbers
Positive Offline Training Samples	Self-captured road image data, Caltech99 and Malaga	6392 images with around 18,000 vehicles
Negative Training Samples	KITTI dataset	Around 20,000 images of the KITTI training set which do not contain vehicles
Test dataset	KITTI dataset	2000 road images containing 7218 vehicles
